# Mining and Biosynthesis of Bioactive Lanthipeptides From Microorganisms

**DOI:** 10.3389/fbioe.2021.692466

**Published:** 2021-07-29

**Authors:** Caiyun Li, Khorshed Alam, Yiming Zhao, Jinfang Hao, Qing Yang, Youming Zhang, Ruijuan Li, Aiying Li

**Affiliations:** ^1^Helmholtz International Lab for Anti-infectives, Shandong University-Helmholtz Institute of Biotechnology, State Key Laboratory of Microbial Technology, Shandong University, Qingdao, China; ^2^State Key Laboratory of Genetic Engineering, School of Life Sciences, Fudan University, Shanghai, China

**Keywords:** natural product, lanthipeptide, biosynthesis, genome mining, pathway engineering

## Abstract

Antimicrobial resistance is one of the most serious public health issues in the worldwide and only a few new antimicrobial drugs have been discovered in recent decades. To overcome the ever-increasing emergence of multidrug-resistant (MDR) pathogens, discovery of new natural products (NPs) against MDR pathogens with new technologies is in great demands. Lanthipeptides which are ribosomally synthesized and post-translationally modified peptides (RiPPs) display high diversity in their chemical structures and mechanisms of action. Genome mining and biosynthetic engineering have also yielded new lanthipeptides, which are a valuable source of drug candidates. In this review we cover the recent advances in the field of microbial derived lanthipeptide discovery and development.

## Introduction

With the emergence of multidrug-resistant (MDR) pathogens, antimicrobial resistance for many clinically-used antibiotics, even the most famous daptomycin, has been reported (Heidary et al., 2018). An increasing and urgent demand for new antibiotics arises clinically. Nevertheless, the frequent rediscovery of known compounds triggered a drastic decrease in new antibiotic detection using traditional drug discovery pipelines and only around 30 antibiotics were approved as new drugs during the last two decades (Weber, 2016; Butler et al., 2017).

Serving as the last line of defense against MDR pathogens, most of peptide antibiotics target highly stable cell walls or cell membranes of bacteria and it is widely considered not easy to develop antimicrobial resistance for peptide antibiotics. Several new important antibiotics reported in recent years, such as cadasides, malacidins and teixobactin, are all peptides with defense capability against a variety of MDR bacteria and without antimicrobial resistance by far (Ling et al., 2015; Guo et al., 2018; Hover et al., 2018; Wu et al., 2019).

**FIGURE 1:**
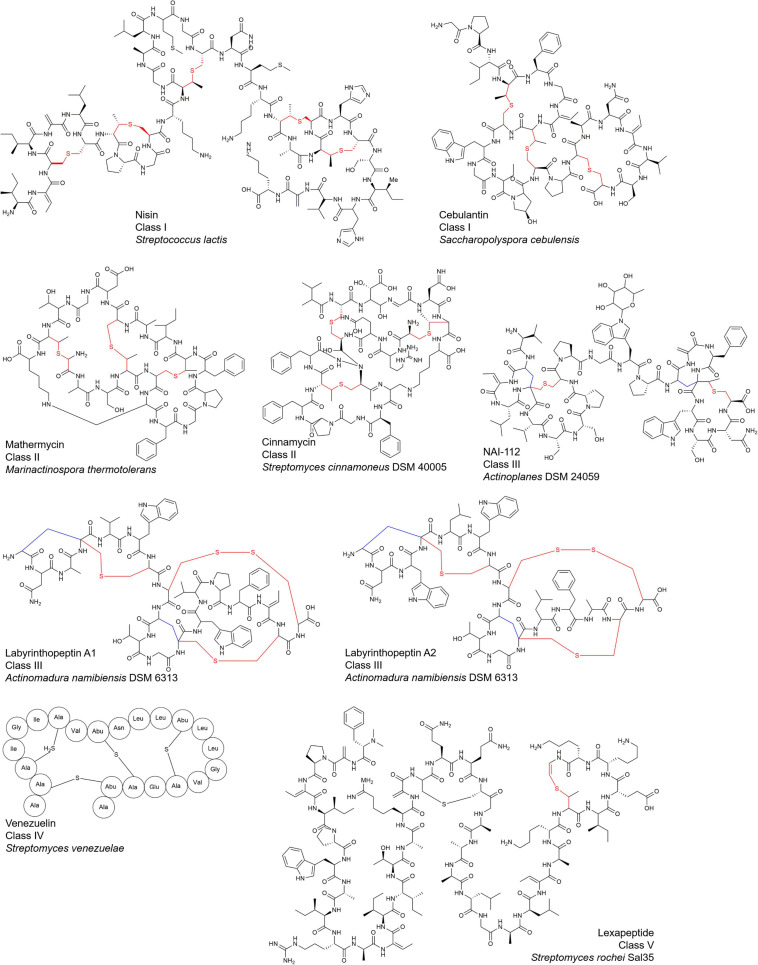
Structures of representative lanthipeptides.

Lanthipeptides, the largest sub-family of ribosomally synthesized and post-translational modification modified peptides (RiPPs) (Arnison et al., 2013), are characterized by the presence of multiple lanthionine (Lan) or (methyl-) lanthionine rings (MeLan) to form thioether bonds (Figure 1). Most of them possess multiple antibacterial mechanisms, endowing them with a high potential in the development of anti-infective drugs (Mo et al., 2016). Nisin (Figure 1), as the first-identified lanthipeptide containing 34 amino acid residues, has been used as a preservative food additive for over 60 years in more than 80 countries, but so far, few nisin-resistant bacteria have been found (Mo et al., 2016; van Staden et al., 2021).

Lanthipeptide biosynthetic gene clusters (BGCs) are widespread within the genomes of microorganisms, providing a substantial repository for novel bioactive peptides. In order to utilize lanthipeptide BGCs, especially, those silent or not accessible in native producers (Zhang et al., 2015), genome-mining (genome-based predication and functional identification of BGCs) has become a commonly used technique in recent years to explore lanthipeptide-derived drugs (Huo and van der Donk, 2016; Chu et al., 2020; Montalbán-López et al., 2021).

## Structures, Microorganism Producers and Bioactivities of Lanthipeptides

Since the discovery of nisin in 1928, about a little more than one hundred of lanthipeptides have been reported from microorganisms, and they display complex and highly diverse structures and bioactivities (Figure 1; van Staden et al., 2021).

### Structural Diversity of Lanthipeptides

Significant difference in peptide chain lengths, amino acid composition of primary sequences, location of thioether bonds, types of unnatural amino acids, and other various structural modification generated a high degree of structural diversity of lanthipeptides:

Lanthipeptides contain varying numbers of characteristic lanthionine (Lan) or methyllanthionine (MeLan) residues (Figure 1), both of which contain a characteristic thioether bond (thioether ring or thioether bridge) formed by linking dehydrated serine or threonine residues (Ser/Thr) to the thiol group of cysteine residues (Cys). The topology of some lanthipeptides containing multiple thioether rings exhibits either linear forms where thioether rings are non-interlaced spatially or complex interlaced ring patterns. Thioether bonds create more stable structures with improved pharmacodynamic properties by protecting peptides from proteolytic degradation (Mo et al., 2016). Besides Lan and MeLan, most lanthipeptides also contain two unnatural amino acid residues including dehydrobutyrine (Dhb) and dehydroalanine (Dha) from dehydrated serine or threonine residues (Ser/Thr).

**FIGURE 2:**
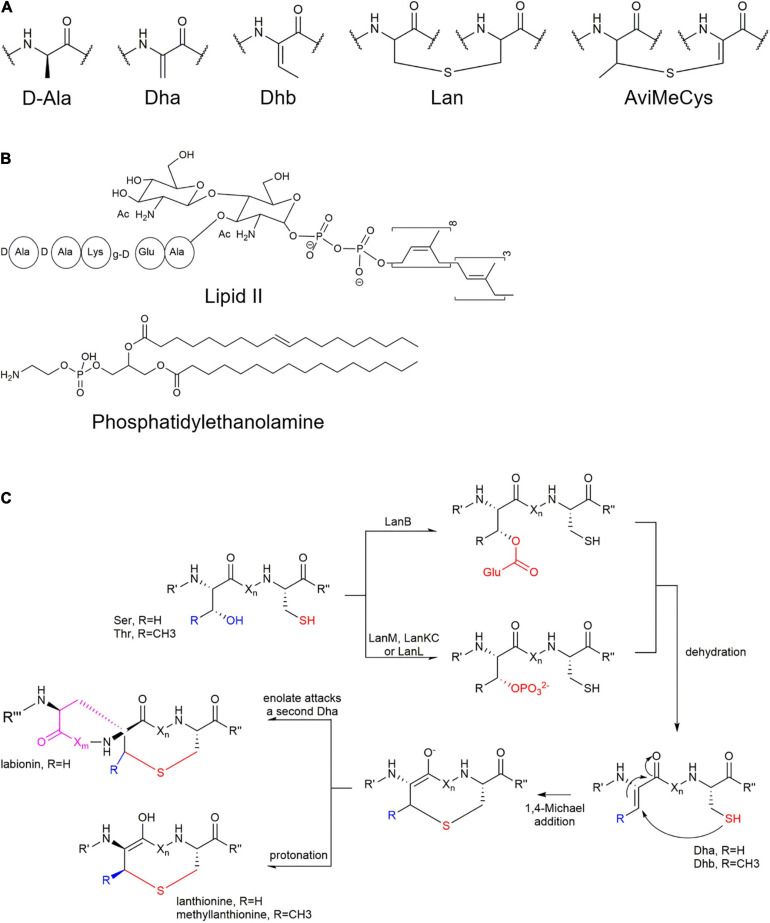
Formation of unusual amino acid residues and action targets of lanthipeptides. **(A)** Unusual amino acid residues in the lanthipeptides. **(B)** Two main action targets of lanthipeptides. **(C)** Formation of characteristic lanthionine (Lan) or methyllanthionine (MetLan). Firstly, Ser/Thr-OH is dehydrated and followed by Cys-SH nucleophilic attack and cyclization. In class I lanthipeptide, Ser/Thr-OH is activated by glutamination at the presence of tRNA^Glu^, followed by deglutamination and dehydration while in class II-IV, Ser/Thr-OH is phosphorylated by LanM/LanKC/LanL and then undergoes dephosphorylation and dehydration. In addition, the labionin in the class III structure is formed by the nucleophilic attack of another Dha enol anion (Ren et al., 2020). It is speculated that class V is also dehydrated through phosphorylation and dephosphorylation mechanisms.

Furthermore, many lanthipeptides contain special AviCys (*S*-[(Z)-aminovinyl]-D- cysteine) structures at the C-terminus (Mo et al., 2016, 2019; Lagedroste et al., 2020; Figures 1, 2). The tailoring structural modification further increase structural diversity of lanthipeptides, such as glycosylation, hydroxylation, halogenation, decarboxylation, acylation, disulfide bond formation, and α-C configuration transformation on some specific amino acid residues (Mo et al., 2016; Figure 2A).

These structural features determine the structural diversity and biological activity of lanthipeptides (Biswas et al., 2017; van Staden et al., 2021; Figures 1, 2).

### Microorganism Producers of Lanthipeptides

Lanthipeptides have been found to accumulate in a variety of microorganisms, and bacteria remain to be the major producers for lanthipeptides (Figure 1 and Table 1).

**TABLE 1:** Producers, bioactivity and BGCs of representative lanthipeptides.

Compound names	Microorganism producers	Bioactivity	Size of BGC	References
Nisin (class I)	*Streptococcus lactis*	Antibacterial and antitumor activity	15 kb	Shin et al., 2016
Subtilin (class I)	*Bacillus subtilis* ATCC 6633	Antibacterial activity	21 kb	Spieß et al., 2015
Microbisporicin (NAI-107) (class I)	*Microbispora corallina*	Antibacterial activity (G^+^ MDR pathogens)	37 kb	Foulston and Bibb, 2010; Ortega et al., 2016; Repka et al., 2017
Cebulantin (class I)	*Saccharopolyspora cebuensis*	Antibacterial activity (G^+^)	10 kb	Moon et al., 2019
Penisin (class I)	*Paenibacillus ehimensis* A3	Antibacterial activity (G^–^ bacteria)	10 kb	Baindara et al., 2015
Pinensins (class I)	*Chitinophaga pinensis*	Antifungi and antiyeast activity	24 kb	Caetano et al., 2020
Duramycin (class II)	*Streptoverticillium Cinnamoneus* ATCC 12686	Antibacterial, antifungi antiviruses/reducing cystic fibrosis and blood pressure, regulating immunity	23 kb	Huo and van der Donk, 2016
Roseocin (class II)	*Streptomyces roseosporus*	Antibacterial activity (G^+^)	23 kb	Singh et al., 2020
Mathermycin (class II)	*Marinactinospora thermotolerans*	Antibacterial and antitumor activity	8 kb	Cheng et al., 2021
Bicereucin (class II)	*Bacillus cereus* SJ1	Antibacterial activity (G^+^ MDR) hemolytic activity.	20 kb	Huo and van der Donk, 2016
Mersacidin (class II)	*Bacillus* sp. strain HIL Y-85,54728	Antibacterial activity (G^+^ MDR)	13 kb	Schmitz et al., 2006
Cinnamycin (class II)	*Streptomyces cinnamoneus* DSM 40005	Antibacterial and antifungus activity	17 kb	Ökesli et al., 2011
Deoxyactagardine B (class II)	*Actinoplanes liguriae* NCIMB41362	Antibacterial activity	35-40 kb	Boakes et al., 2010
Haloduracin (class II)	alkaliphile *Bacillus halodurans* C−125	Antimicrobial activity	27 kb	Lawton et al., 2007
Landornamide A	*Kamptonema* sp. PCC 6506	Antiviral activity	12 kb	Bösch et al., 2020
Labyrinthopeptin (class III)	*Actinomadura namibiensis* DSM 6313	Antiviral activity	6 kb	Rupcic et al., 2018
NAI-112 (class III)	*Actinoplanes* DSM 24059	Antipyretic analgesic	–	Monciardini et al., 2014; Repka et al., 2017; Chen et al., 2019
Sfl (class IV)	*Streptomyces* sp. NRRL S-1022	–	–	Ren et al., 2020
Lexapeptide (class V)	*Streptomyces rochei* Sal35	Antibacterial activity (G^+^)	28 kb	Xu et al., 2020

Actinobacteria: Lanthipeptides were identified with the majority in Actinobacteria (Gomes et al., 2017) and genome sequencing revealed that lanthipeptide BGCs are extremely abundant in the genomes of Actinobacteria (Vikeli et al., 2020; Walker et al., 2020). Several examples reported in recent years include lexapeptide isolated from *Streptomyces rochei* Sal35 (Xu et al., 2020), roseocin isolated from *Streptomyces roseosporus* (Singh et al., 2020), *mathermycin* isolated from *Marinactinospora thermotolerans* (Chen et al., 2017), cebulantin derived from *Saccharopolyspora cebuensis* (Moon et al., 2019), microbisporicin obtained from *Microbisporus corallina* (Fernández-Martínez et al., 2015) and so on.

Firmicutes: Firmicutes are also a large source for lanthipeptides, among which, *Bacillus* species were identified as notably producers. As an example, bicereucin was produced by *Bacillus cereus* (Huo and van der Donk, 2016). Nisin was firstly isolated from *Streptococcus lactis*, and its analogs have been found in many kinds of bacteria. Recently, it has been discovered that nisin analogs could be derived from *Staphylococcus capitis* residing in human skins (O’Sullivan et al., 2020).

Bacteroides: wide distribution of lanthipeptides was also observed in Bacteroides (Walker et al., 2020). For instance, abundant lanthipeptide BGCs have been found in the genomes of *Chryseobacterium*, *Pedobacter* and *Flavobacterium*. Multiple lanthipeptides including pinensins have been found in *Chitinophaga pinensis* (Caetano et al., 2020).

Besides general bacteria, cyanobacteria and even archaea also are attracting more attentions in recent years as potential producers of lanthipeptides. Exemplified are prochlorosins, a group of lanthipeptides, which were produced by certain strains of the ubiquitous marine picocyanobacteria, like species in *Prochlorococcus* and *Synechococcus*. And picocyanobacteria are found to produce remarkably thousands of different cyclic peptides, few of which would display similar ring topologies (Cubillos-Ruiz et al., 2017). Landornamide A synthesized by a silent by BGC from a cyanobacterium *Kamptonema* sp. PCC 6506 was identified via pathway reconstruction in *E. coli* (Bösch et al., 2020). Archaea might be producers for lanthipeptides because a high number of lanthipeptides gene clusters were detected (Walker et al., 2020).

### High Diversity of Bioactivities of Lanthipeptides

Most of known lanthipeptides have antibacterial activity, especially against MDR strains, while some have antiviral, antitumor, antifungal, and immunomodulatory properties and some display the potential to alleviate cystic fibrosis symptoms. Other activities, such as morphogenetic and antinociceptive actions, have also been reported (van Staden et al., 2021; Table 1).

For instance, microbisporicin, NAI-107, mersacidin and penisin exhibit inhibition against antibiotic-resistant pathogens, such as methicillin-resistant *Staphylococcus aureus* (Baindara et al., 2018). Cinnamycin could kill *Bacillus* and also has antifungal ability (Chen et al., 2017; Vestergaard et al., 2019). Cebulantin is effective against the growth of various *Vibrio* pathogens (Moon et al., 2019). Deoxyactagardine B derivatives have entered clinical trials as antibacterial infection drugs. Nisin, as the first food additive, has a strong inhibitory effect on many G^+^ bacteria including *Streptococcus pneumoniae*, and also has certain inhibitory activity on some G^–^ bacteria (Ahmadi et al., 2017; Li et al., 2018). However, compared to the effects against G^+^ bacteria, most of lanthipeptides show relatively weak inhibitory activity against G^–^ bacteria (Liu et al., 2020).

In addition to antibacterial activity, nisin has inhibitory effects on head-neck squamous cell carcinoma (Ahmadi et al., 2017). Mathermycin shows inhibitory activity on non-small cell lung cancer and other tumors (Cheng et al., 2021). Bicereucin possesses hemolytic activity (Huo and van der Donk, 2016). Labyrinthopeptin is effective against a variety of viral infections caused by HIV, hepatitis C virus, herpes simplex virus, and respiratory syncytial virus (Prochnow et al., 2020). NAI-112 has been proved to reduce inflammation and relieve pain (Iorio et al., 2014). Duramycin is particularly effective in the treatment of cystic fibrosis, and some lanthipeptides have other properties such as lowering blood pressure or regulating immunity (Ahmadi et al., 2017).

## Therapeutic Action Mechanisms of Lanthipeptides

It has been proposed that the target affinity of lanthipeptides is of great importance and crucial for their therapeutic potential (van Staden et al., 2021).

Most lanthipeptides exhibit antibacterial activity by binding to lipid II, an essential intermediate in peptidoglycan biosynthesis (Figure 2B) to result in blocking of cell wall biosynthesis and disruption of the cell membrane integrity. Taking nisin as an example, compared to vancomycin, nisin has a different binding site with lipid II. The two thioether rings of nisin can bind to the pyrophosphate group of lipid II to form a cage-like complex. In addition, its binding with lipid II leads to the formation of a polymerized complex, which causes cell membrane perforation and electrolyte outflow (Dickman et al., 2019). The essential role of cyclic structures of nisin for lipid II-binding proposed that such constrained structures caused by thioether rings imposed on lanthipeptides is suitable or required for target binding (Bosma et al., 2019). As one of the peptidoglycan precursors, the target lipid II is not prone to mutate to reduce resistance to lanthipeptides.

Many lanthipeptides exert bioactivity by recognizing and combining phosphatidylethanolamine which is an important component of membrane structures in for instance cell membranes and viral envelopes (Figure 2B). The antiviral labyrinthopeptin A1 was proved to bind to the specific motif of phosphatidylethanolamines in the virus envelope, leading to the lysis of the virus and interfering with the structural integrity of the virus envelope (Blockus et al., 2020; Prochnow et al., 2020). Cinnamycin with bactericidal activity can target the primary amino group of phosphatidylethanolamine on the bacterial cell membrane to form a hydrogen-bonding complex. This complex could also specifically bind to the phosphate group of phosphatidylethanolamines (Vestergaard et al., 2019). Since the expression of phosphatidylethanolamines on the inner and outer surfaces of tumor cell membranes is abnormally abundant, mathermycin is able to interfere with the metabolic activity of tumor cells and induce tumor cell necrosis by targeting phosphatidylethanolamines (Cheng et al., 2021; van Staden et al., 2021).

The two-component synergistic mechanism is a special mode for certain lanthipeptides. Unlike most lanthipeptides exerting their biological activity through a single peptide chain component, the bioactivity of a small but increasing number of lanthipeptides relies on synergistic action of two components in a 1:1 ratio while the single component has no or only very weak activity. Taking haloduracin α and haloduracin β from alkalophilic *Bacillus* as an example, two peptide chains and lipid II are combined in a 2:2:1 ratio to form a complex, which inhibits cell wall biosynthesis and mediates cell membrane perforation (McClerren et al., 2006; Oman et al., 2011). Bicereucin, roseocin and lacticin 3147 also showed synergistic antibacterial activity against G^+^ bacteria at a ratio of 1:1 in a two-component mode, probably by targeting the formation of the cell wall (Martin et al., 2004; Huo and van der Donk, 2016; Singh et al., 2020). It has been revealed recently that combination between labyrinthopeptin A1/A2 could significantly improve its antiviral activity (Figure 1; Blockus et al., 2020).

It is noteworthy that some lanthipeptides could target other targets. The sugar transport system (sugar PTS proteins) on the cell membranes is also the target for some lanthipeptides, which display cell wall lysis activity by activating *N*-acetyl-1-alanine amidase and *N*-acetylglucosaminidase. Furthermore, some lanthipeptide antibiotics are capable of binding to cell mitochondrial membranes, leading to cell autophagy (Zhang and Liu, 2013; Sandiford, 2019), and some lanthipeptides, such as nisin and subtilin, were found to covalently modify the targets on the spore wall of *Bacillus* to inhibit spore germination. In recent years, it has also been reported that nisin may interfere with DNA replication, recombination and repair in *E. coli*, though the mechanism has yet to be elucidated (Galván Márquez et al., 2020).

## Biosynthesis Mechanism and Classification of Lanthipeptides

**FIGURE 3:**
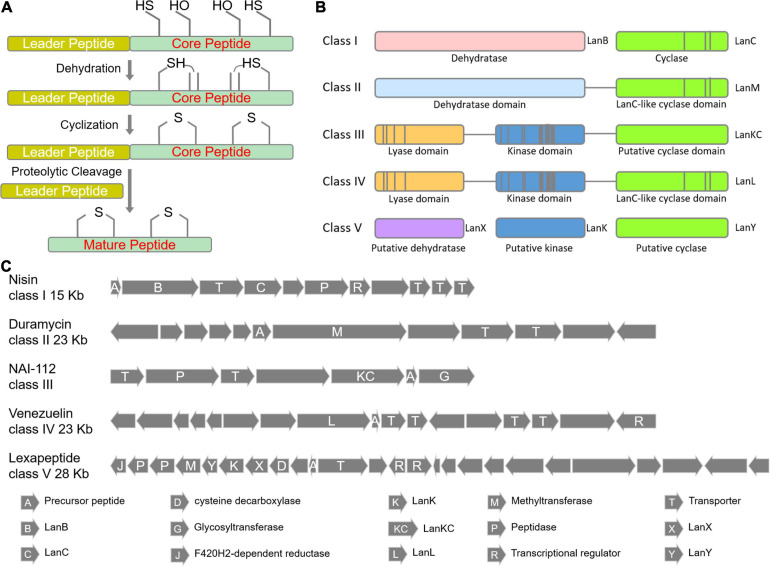
Biosynthetic pathways of lanthipeptides. **(A)** The general steps of lanthipeptide biosynthesis. **(B)** Five classes of key enzymes for the formation of thioether ring. **(C)** Representative BGCs for lanthipeptide biosynthesis.

The biosynthesis of lanthipeptides were generally divided into four stages (Figures 2, 3): (i) synthesis of small molecule precursor peptides directed by ribosomes; (ii) formation of thioether rings (dehydration and ring formation); (iii) other post-translational modifications, and (iv) removal of the N-terminal leader peptides. The formation of the thioether ring requires the presence of the leader peptides (Figure 2C and Figure 3A). The common enzymes encoded by lanthipeptide BGCs include the precursor peptide synthetase (LanA), key enzymes (such as LanB, LanC and so on) for dehydration and cyclization to catalyze the formation of the thioether rings (Figure 2C), the protease (LanP) for cleaving the leader peptide and LanT for peptide transport (Figure 2C). In addition, some lanthipeptide BGCs also encode some special enzymes for tailoring modification, such as glycosylase, oxidase, methyl transferase and decarboxylase. According to the different key enzymes that catalyze dehydration and cyclization required for the synthesis of thioether rings, lanthipeptides can be divided into five classes at present (Figures 1–3).

As shown in Figure 3B and Figure 3C, lanthipeptides in Class I and Class II are more frequently identified, most of which show antibacterial properties. Both dehydratase LanB and cyclase LanC are single-functional separate enzymes for class I while LanM has dual functions for dehydration and cyclization for class II. LanB for class I is characterized by its role to activate serine and threonine residues by glutamylation in a tRNA-dependent manner (Zhang and Liu, 2013; Figure 2C). LanKC for class III and LanL for class IV have different key motif regions, though both of them are multifunctional enzymes and catalyze dehydration using dedicated kinase and lyase domains to perform similar functions (cleavage, phosphorylation and cyclization) (Figure 2C; Müller et al., 2013). Unlike lanthipeptides in class I and II, class III and IV lanthipeptides are quite rare, most of which possess other actions than antibacterial activity (Goto et al., 2010). Lexapeptide A as first member in class V by far was identified in 2020 (Figure 1). Its BGC encodes three single-functional key enzymes responsible for phosphorylation, dehydration and cyclization respectively, remarkably different from class I-IV (Xu et al., 2020; Figure 2C). Deciphering of NCBI Datasets for these key enzymes encoded by class V BGCs demonstrated a high degree of diversity in their structures and catalytic mechanisms. Their classification might be far more complex than those in class I-IV, implying the rich structural and biosynthetic diversity of class V lanthipeptides in nature (Walker et al., 2020; Xu et al., 2020).

A structural view on the maturation of lanthipeptides concerning the mechanisms of these key enzymes (class I-IV) was well reviewed (Lagedroste et al., 2020).

Compared to relatively clear biosynthetic mechanisms of key enzymes in class I-II, those in class III-V possess some unusual features yet to be elucidated in the terms of structure and biosynthesis.

The following are three typical biosynthetic enzymatic systems newly reported for insight into comparatively rare class III-V lanthipeptides:

(i) Class III: NAI-112 contains two labionin macrocyclic structures. Labionin is a characteristic feature for class III lanthipeptides and its formation is mediated by three amino acid residues (Ser-Ser/Thr-Cys) which undergo dehydration and cyclization by two steps of Michael addition reactions to form a thioether ring and a carbocyclic ring (Figure 2C; Müller et al., 2013). The formation of such labionin macrocycles in NAI-112 is catalyzed by multifunctional AplKC. A rare *N*-deoxyhexose glycosylation was identified on Try residue of NAI-112 and completed by *N*-glycosyltransferase AplG. AplP, as a broad-substrate-spectrum protease with dual functions, is responsible for the cleavage of the leader peptide of NAI-112 (Iorio et al., 2014; Chen et al., 2019; Sheng et al., 2020; Figure 3C).
(ii) Class IV: Modified-SflA isolated from *Streptomyces* contains two non-interlaced thioether rings. Although its BGC contains no aminopeptidase gene, it was found that an unknown aminopeptidase gene outside of its BGC can assist the excision of the leader peptide. The cyclization process was revealed to occur simultaneously at multiple sites of its core peptide (Ren et al., 2020).
(iii) Class V: Lexapeptide has a thioether ring formed by three independent modification enzymes, LxmK, LxmX and LxmY. It also contains several rare amino acid residues, such as N-terminal (*N*, *N*)-dimethyl-Phe and C-terminal (2-aminovinyl)-3-methyl-Cys and *D*-Ala. The formation of its rare D-Ala was catalyzed by a novel coenzyme F420-dependent oxidoreductase LxmJ, which is responsible for the reduction of dehydrated alanine to form this unnatural amino acid (Xu et al., 2020; Figure 3C).

## Mining and Biosynthetic Reconstruction of New Lanthipeptides

Though the number of lanthipeptides uncovered is still small, they are attracting more attentions in solving MDR problem. Analysis of expanding bacterial genome sequencing data revealed that a great wealth of lanthipeptide BGCs present in microorganisms have become important resources for exploration of new lanthipeptides, despite of that a majority of these BGCs are silent, expressed inefficiently in native strains, or present in unculturable strains or in metagenomic samples (Blin et al., 2014).

Genome-mining has become a promising solution for NP discovery and broadened the concept of NP producers. It involves (i) predication *in silico*, (ii-a) cloning and heterologous expression or (ii-b) activation *in situ* of previously uncharacterized BGCs, followed by experimental identification of the products of the gene clusters and further (iii) biosynthetic pathway engineering to produce derivative products with higher productivity or improved therapeutic actions (Figure 3; Abbasi et al., 2020).

### *In silico* Analysis of Lanthipeptides BGCs

Various bioinformatics methods such as NCBI BLAST, antiSMASH, RiPPMiner, PRISM, RiPP-PRISM, RODEO, BAGEL, Natural product peptidogenomics (NPP) and RiPPquest, have been developed for lanthipeptide discovery (An and van der Donk, 2020).

Among them, RODEO (Rapid ORF Description and Evaluation Online) and antiSMASH (antibiotics and secondary metabolite analysis shell) are most commonly used tools to identify BGCs via large-scale mining of RiPP data (Tietz et al., 2017).

An expanded RODEO was used to search the RefSeq database, 8405 precursors peptide synthase (LanA) were predicted *in silico* successfully, though *lan*A identification has been a challenge due to their variability and small size often not annotated as genes. By searching non-redundant RefSeq database (release 93) with LanC domain shared in all known classes of lanthipeptides and BGC categorization, plenty of lanthipeptide BGCs were identified, including 2753 putative class I lanthipeptide BGCs, 3708 class II BGCs, 2377 class III BGCs, 815 class IV BGCs. Application of this approach revealed that lanthipeptide BGCs are distributed widely across bacterial phyla (Tietz et al., 2017).

antiSMASH is a use-friendly tool for mining of lanthipeptide BGCs by simply submitting genome sequences into this NP BGC analytic platform.^1^ Many lanthipeptide BGCs have been identified using antiSMASH (Arnison et al., 2013; Blin et al., 2014). The current version of the antiSMASH database contains annotations for 6200 full bacterial genomes and 18,576 bacterial draft genomes (Blin et al., 2019a,b), although antiSMASH does not distinguish between class III and class IV BGCs.

### Functional Identification of Lanthipeptide BGCs

Generally, cultural condition optimization still seems quite useful for a supportive and first- trial method to accelerate accumulation of lanthipeptide of interest (Li et al., 2010; Cubillos-Ruiz et al., 2017). Otherwise, BGC direct capture or pathway reconstruction and heterologous expression or *in situ* activation are required in most of cases (Table 2).

**TABLE 2:** Strategies for mining of lanthipeptides.

Strategies	Gene origins	Compound classification	Compound example	References
RODEO+ expression *in situ* (optimized culture conditions)	*non*-redundant *Ref*Seq database and *Streptomyces rimosus*	Class III and IV	Birimositide α and β	Walker et al., 2020
BAGEL3+ direct cloning and heterologous expression	*Corynebacterium lipophiloflavum* DSM 44291 and *Streptococcus agalactiae* ATCC 13813	Class I and II	Flavucin and agalacticin	van Heel et al., 2016
DeepRiPP+genomic and metabolomic data+expression *in situ*	*Flavobacterium ginsengiterrae* JCM 17337, *Chitinophaga* sp. CHO1, and *Chitinophaga* sp.	–	Deepgensin deepflavo	Merwin et al., 2020
Direct cloning and heterologous expression platform *or in situ* activation	*Streptomyces formicae* KY5	Class II	Kyamicin	Vikeli et al., 2020
Expression *in situ* (optimized culture conditions)	*Prochlorococcus* and *Synechococcus*	Class II	Prochlorosins	Li et al., 2010; Cubillos-Ruiz et al., 2017
Pathway reconstruction and heterologous expression	*Kamptonema* sp. PCC 6506	Class II	Landornamide A	Bösch et al., 2020
LEXAS	*Streptomyces rochei* Sal35	Class V	Lexapeptide	Xu et al., 2020
CFPS	NCBI database	Class I	Nisin derivatives	Zhang et al., 2018; Liu et al., 2020

**FIGURE 4:**
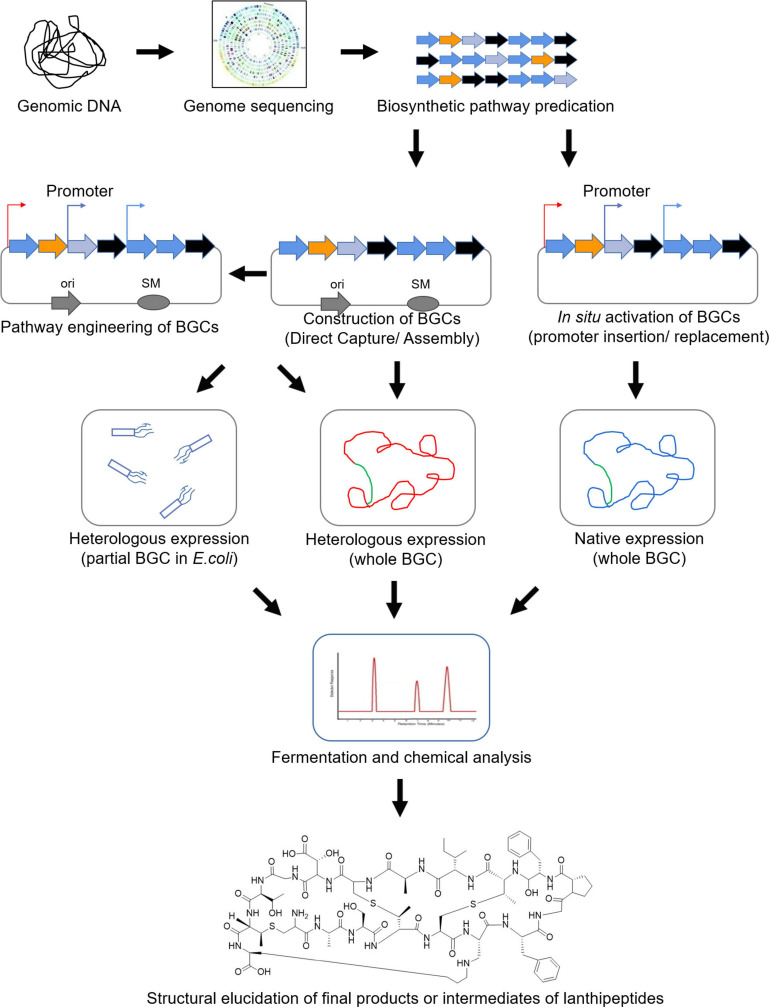
Scheme of genome mining of lanthipeptides.

To capture the whole lanthipeptide BGCs, TAR (transformation-assisted recombination) and Recombineering (Red/ET-mediated recombination engineering) have been developed into most commonly-used cloning techniques. Based on the indigenous yeast recombinases, TAR is capable of one-step acquisiting NP BGCs larger than 50 kb in size from microorganism genomes, or used for reconstruction of BGCs from the environmental DNA libraries. Recombineering based on phage recombinases Red/ET displays advantageous potential in mining of NP BGCs in size up to 106 kb (Wang et al., 2018; Abbasi et al., 2020). Recently, combining CRISPR/Cas9-derived systems with TAR or Recombineering has proved to improve significantly the efficiency in direct cloning, refactoring and heterologous expression of NP BGCs (Wang et al., 2018; Figure 4).

Using these strategies in recent years, some new lanthipeptides have been identified by cloning or reconstructing their biosynthetic pathways and expressing them in heterologous hosts, while some lanthipeptides were identified by inserting strong promoters into the genome in the original bacteria to activate the silent BGCs of these compounds (van Heel et al., 2016; Bobeica and van der Donk, 2018; Hegemann and van der Donk, 2018; Huo et al., 2019; Bösch et al., 2020; Sandiford, 2020; Table 2).

In addition, some high-throughput genome-mining strategies have been established for discovery of lanthipeptides, as demonstrated, for instance, by LEXAS (library expression analysis system). LEXAS was used to successfully identify Streptomyces-derived lexapeptide as the first example in class V lanthipeptide, highlighted with high-throughput mobilization of BAC clones into *Streptomyces* hosts and high-throughput bioassay for antimicrobial activity (Xu et al., 2016, 2020). Many lantipeptide display systems were ever established to allow preparation of different lanthionine-containing peptides, including *in vitro* display systems using RNA/phage and *in vivo* display systems using bacterial/yeast (Hofmann et al., 2012; Bosma et al., 2019). Currently, some *in vitro* cell free biosynthesis platforms were well-designed for mining of lanthipeptides with the superiority of independent of cell growth, especially combined with high-throughput bioactivity screening systems. For instance, by use of CFPS (cell-free protein synthesis) set up based on *E. coli* cell extract, searching all nisin-related gene sequences in NCBI database resulted in biosynthesis of four new lanthipeptides with antibacterial activity in a single day (Zhang et al., 2018; Liu et al., 2020).

### Biosynthetic Pathway Engineering of Lanthipeptides

Compared with other types of antibiotic BGCs, it is easier to carry out genetic manipulation on lanthipeptide BGCs due to their smaller size (<30 kb), which facilitates pathway engineering of lanthipeptide BGCs to yield new bioactive products (Table 2).

Precursor peptide synthase (LanA) was a common target for pathway engineering. For example, Schmitt S group divided these enzymes from biosynthetic pathways of 12 natural lanthipeptides into 33 modules which differs in sites of thioether rings and target binding, and performed “combinatorial shuffling” of these modules at the DNA level. Finally, they obtained the synthesis of 6,000 derived precursor peptides. Then heterologous expression by combining with the post-translational modification system for nisin and a high-throughput micro-screening system led to identification of 11 new lanthipeptides with enhanced antibacterial activity (Schmitt et al., 2019).

Some studies also focus on the engineering of key enzymes for thioether ring formation. For instance, in 2019, Kuipers OP group fused the Ser/Thr-dehydratase NisB and the cyclase NisC for nisin production to the biosynthetic pathway of vasopressin, and obtained a vasopressin analog with replacement of an original disulfide bridge with a thioether bridge, providing the proof for applying the post-translational modification systems of lanthipeptides into generation of new bioactive products (Li et al., 2019).

In addition, in order to enhance the outer membrane-traversing efficiency of nisin, by fusing the C-terminal of nisin with several relatively short peptides with activity against G^–^ bacteria reported in references, a derivative compound was generated with increased activity by 4- to 12-fold compared to that of nisin, when inhibiting against several important G^–^ pathogens, including *Escherichia coli*, *Klebsiella pneumoniae*, *Acinetobacter baumannii*, *Pseudomonas aeruginosa* and *Enterobacter aerogenes* (Li et al., 2018).

### Structural Determination of Lanthipeptides

Generally, similar to other type NPs, most commonly used approaches for structures elucidation of newly discovered lanthipeptides include nuclear magnetic resonance (NMR) spectroscopy, mass spectrometry (MS) and some new approaches (Leenders et al., 2015; Wang et al., 2016).

To determine the thioether ring topology of new lanthipeptides, NMR spectroscopy is the most reliable approach, if regardless of its longer operation and requirements for considerable quantities of target products. To distinguish multiple overlapped rings in lanthipeptides, some new strategies based on mass spectrometry, such as Matrix-assisted laser desorption/ionization time-of-flight mass spectrometry (MALDI-TOF-MS) combined with LIFT tandem MS sequencing or LC-ESI-MS/MS have been established to fragment modified LanAs (Zhang et al., 2014; Tang et al., 2015; Schrimpe-Rutledge et al., 2018).

To confirm the formation and stereochemistry of Lan or MeLan, modified peptides (LanAs) are typically hydrolyzed under acidic conditions into the individual amino acids and reduced to yield disulfide bonds. Then the hydrolyzed mixtures containing free Cys thiols are derivatized into more volatile components with mass change, then analyzed by gas chromatography mass spectrometry (GC-MS) (An and van der Donk, 2020).

It is notable that Global Natural Product Social Molecular Networking (GNPS) as a generic metabolomics portal to analyze the MS/MS data would likely be used for structural analysis of lanthipeptides (Wang et al., 2016).

## Conclusion and Prospects

Over the past years, tremendous advances have been reported in the understanding of lanthipeptides at the levels of genetics, bioactivity, structure and enzymology.

Though the number of known lantibiotics is still limited, it is still greatly advantageous to explore anti-infective drugs from lanthipeptides, due to the rarely observed antimicrobial resistance and abundance of their microbial-derived BGCs in nature. Using genome-mining, it is expected to isolate and identify new bioactive lanthipeptides from microorganisms.

By far, most of lanthipeptides with antibacterial activity have displayed very potent activity against G^+^ bacteria. But their activity against G^–^ bacteria is relatively lower. Precise engineering lanthipeptide available at DNA levels could be an applicable solution to get new lanthipeptides against G^–^ bacteria.

Compared to class I-II, biosynthesis of most of class III-V lanthipeptides still remains unclear and their activity other than antibacterial actions highlighted the importance of their potential value as new antiviral or antitumor agents. More insights into the novel mechanisms of their post-translational modification systems will be considerably required, especially in the molecular coordination and timing of the maturation enzymes and their interplay with the exporter proteins. As well, it is of importance to precisely engineer their biosynthetic pathways to enhance their activity and production yields.

## Author Contributions

CL and AL: draft writing. KA, YiZ, and JH: figure drawing. YoZ and RL: draft planning and organization. AL: draft organization and manuscript writing. All authors contributed to the article and approved the submitted version.

## Conflict of Interest

The authors declare that the research was conducted in the absence of any commercial or financial relationships that could be construed as a potential conflict of interest.

## Publisher’s Note

All claims expressed in this article are solely those of the authors and do not necessarily represent those of their affiliated organizations, or those of the publisher, the editors and the reviewers. Any product that may be evaluated in this article, or claim that may be made by its manufacturer, is not guaranteed or endorsed by the publisher.

## Footnotes

1
https://antismash.secondarymetabolites.org/
